# The effectiveness of substitution of hospital ward care from medical doctors to physician assistants: a study protocol

**DOI:** 10.1186/1472-6963-14-43

**Published:** 2014-01-28

**Authors:** Marijke JC Timmermans, Anneke JAH van Vught, Michel Wensing, Miranda GH Laurant

**Affiliations:** 1 Scientific Institute for Quality of Healthcare, Radboud University Medical Center, Nijmegen, The Netherlands; 2 Master Physician Assistant, HAN University of Applied Sciences, Nijmegen, The Netherlands; 3 Faculty of health, behavior and society, HAN University of Applied Sciences, Nijmegen, The Netherlands

**Keywords:** Substitution, Task reallocation, Hospitalist, Physician assistant, Costs, Quality

## Abstract

**Background:**

Because of an expected shrinking supply of medical doctors for hospitalist posts, an increased emphasis on efficiency and continuity of care, and the standardization of many medical procedures, the role of hospitalist is increasingly allocated to physician assistants (PAs). PAs are nonphysician clinicians with medical tasks. This study aims to evaluate the effects of substitution of hospital ward care to PAs.

**Methods/Design:**

In a multicenter matched controlled study, the traditional model in which the role of hospitalist is taken solely by medical doctors (MD model) is compared with a mixed model in which a PA functions as a hospitalist, contingent with MDs (PA/MD model). Twenty intervention and twenty control wards are included across The Netherlands, from a range of medical specialisms. Primary outcome measure is patients’ length of hospital stay. Secondary outcomes include indicators for quality of hospital ward care, patients experiences with medical ward care, patients health-related quality of life, and healthcare providers’ experiences. An economic evaluation is conducted to assess the cost implications and potential efficiency of the PA/MD model. For most measures, data is collected from medical records or questionnaires in samples of 115 patients per hospital ward. Semi-structured interviews with healthcare professionals are conducted to identify determinants of efficiency, quality and continuity of care and barriers and facilitators for the implementation of PAs in the role of hospitalist.

**Discussion:**

Findings from this study will help to further define the role of nonphysician clinicians and provides possible key components for the implementation of PAs in hospital ward care. Like in many studies of organizational change, random allocation to study arms is not feasible, which implies an increased risk for confounding. A major challenge is to deal with the heterogeneity of patients and hospital departments.

**Trial registration:**

ClinicalTrials.gov ID NCT01835444

## Introduction

### Background

Healthcare systems across the world face a number of challenges, such as a rising demand for healthcare services, a growing number of chronic ill patients and rising patient expectations. Concurrently, the supply of medical doctors (MDs) is constrained in most countries, leading to workforce shortages [1]. Nonphysician clinicians have been introduced into the medical domain in order to take over tasks from MDs [2]. An example of a nonphysician clinician is the Physician assistant (PA), a health care professional licensed to practice medicine in defined domains, in collaboration with MDs but with a substantial degree of professional autonomy [3]. PAs obtain medical history, perform physical examinations, request and interpret additional testing, render medical diagnoses and treatment procedures, and prescribe medication. They also perform specific medical procedures, such as endoscopies, catheterizations, elective cardioversion and minor surgeries [3,4]. In addition, PAs contribute to the quality of care by developing protocols, initiate or participate in quality projects and education programs [5].

The PA was first introduced in the sixties in the United States and then rapidly spread across the country [4]. In the Netherlands, the first PAs were introduced in 2001 [6,7]. Currently approximately 630 graduated PAs are employed in the Dutch healthcare system, on a total of about 65 000 registered physicians [8]. In the next few years, about 120 PAs will yearly complete their Master program. Contrary to the USA, where the majority of PAs work in primary care settings, most Dutch PAs (about 75%) work in the hospital settings [9]. The majority works at general surgery, surgical subspecialties, cardiology, anesthesiology or internal medicine [10]. The main features of Dutch PA’s are [7,10]:

• PAs follow a 30 month training program at a Master’s degree level.

• The Dutch PA programs incorporate a dual work-education model, which means that students are employed within a particular medical specialty while enrolled in the master’s PA program. The students undertake didactic and clinical education within this medical specialty from the beginning till the end of the curriculum.

• PA students are professionals with a health care-related bachelor’s degree and at least 2 years of clinical work experience in the health care domain.

• PAs conduct low to moderately complex medical tasks within a certain specialty, both in primary and secondary care. Most PAs practice in the hospital setting.

• Since January 2013, PAs are authorized to indicate and perform predefined medical procedures and subscribe medication without supervision. The scope of practice will be re-evaluated in 2017.

• Physician Assistant is a protected title by law. The legislation is written in the Individual Health Care Professions Act (Wet BIG), article 36a.

Since the first introduction of the PA, several studies have examined their performance. This body of evidence suggests that PAs can provide high-quality care in a large range of medical disciplines [11-14]. The studies indicate that they provide care that is comparable to that of MDs, with high levels of patient satisfaction [15-18]. Although there is international evidence for both efficacy and effectiveness supporting the reallocation of care from MDs to PAs, current research does not cover all settings and professions [2,13]. Many studies concern primary and critical care settings, while studies assessing the effects of substitution of non-acute inpatient medical care are limited. Some studies show methodological limitations like single centered, non-randomized, a relatively small sample size or no control condition. Besides, concerns have been expressed regarding potential adverse effects of involving PAs, such as negative impacts on patient safety and continuity of healthcare delivery.

In this study we focus on patients admitted to a hospital, who are taken care by a hospitalist. Hospitalists are responsible for the coordination of the daily medical care of hospitalized patients [19]. This role has traditionally been fulfilled by medical residents (MRs) and occasionally by medical specialists. In recent years, the role of hospitalist has been increasingly reallocated to PAs [3,11], facilitated by technological innovations and the standardization of many medical procedures by clinical protocols [20,21]. In 2013, approximately 200 graduated PAs were employed as hospitalist in the Netherlands. When PAs are employed as hospitalists, the applied model to cover 24/7 ward care is often a mixed model that contains both PAs and MDs as hospitalist, comprising a patient medical care team. The tasks of PAs in such a team are comparable to those of the MDs. The PAs, however, tend to work during daytime on weekdays, while MDs often work during evenings, nights and weekends. It is anticipated that within the next decades PAs will be increasingly employed in the management of hospitalized patients for a range of different specialism. However, empirical evidence about the consequences of reallocating medical ward care from MDs to PAs for the quality and safety of care is currently limited.

### Study aim

The primary aim of this study is to determine the effectiveness of hospital ward care by MDs compared to a patient medical care team consisting of both PAs and MDs. It is hypothesized that due to reallocation of care to a fixed number of PAs per hospital ward, inpatient care becomes more standardized and continued resulting in improved care, which will be reflected by shorter hospital stay. To measure effectiveness we therefore choose length of hospital stay (LoHS) as primary outcome measure. Besides the effectiveness, also the effects on quality and continuity of care and patient and care provider experiences are investigated.

## Methods/Design

### Study design and population

A multicenter non-randomized matched-controlled study is performed in The Netherlands, comparing wards utilizing a mixed ‘PA/MD model’ (intervention group) with wards utilizing a solely ‘MD model’ (control group, usual care). Control wards are matched with the intervention wards on the basis of medical specialism and hospital type (i.e. academic versus non-academic). Data collection runs parallel for each pair of matched intervention and control ward, with a maximum deviation of two weeks.

### Study setting

Hospital wards are being assigned to the intervention group if the PA has completed an accreditated master’s PA degree and covers at least 51% of the available ward care hours per week during dayshifts (8 h-18 h) on weekdays. Wards are assigned to the control group if solely MDs fulfill the hospitalist position. Exclusion criteria at ward level are: 1) Nurse practitioners (including in training) in the role of hospitalist; 2) Only non-graduated PAs in the role of hospitalist; 3) Psychiatric and pediatric wards and intensive care units. In order to enhance the generalizability of findings we include a heterogeneous sample of hospitals across the country and a mix of medical specialism.

### Study population

The focus of this study is on the patients admitted to the included hospital wards. Exclusion criteria at patient level are: 1) Patients younger than 18 years; 2) Terminally ill patients; 3) Patients in daycare. Daycare is defined as hospital admissions which are intended to last 24 hours or less. For patients who are not able to fill in questionnaires (e.g. patients with cognitive impairment), family relatives are asked to fill in the questionnaires. Besides the patients, also the PAs, MDs, and a sample of ten nurses who are employed at the included ward are involved as study objects. The sample of nurses is established by selecting the first ten nurses who are scheduled for a dayshift during the third week from data collection.

### Primary outcome

LoHS is the primary outcome measure. Reducing LoHs is important for payers of healthcare and for many patients. LoHS is defined as the time period in days between date of discharge and date of admission. To control for discharge delay for nonmedical reasons, i.e. delay attributable to waiting times for a place in a nursing home or a rehabilitation clinic, or help in the patient’s own home, we also register the date of completion of medical treatment in the hospital.

### Secondary outcomes

#### Quality of hospital ward care

To assess the quality of ward care, a set of eleven global clinical and process indicators has been selected from the literature and suggestions by a physician panel. The clinical indicators were derived from a national set of indicators for quality of hospital care from the Dutch Health Care Inspectorate (IGZ) [22]. All indicators cover a period of maximum one month after discharge. The selected indicators are:

Clinical indicators:

• Inhospital mortality

• Unplanned transfer to intensive care unit

• Cardiopulmonary resuscitation

• Pressure sore developed during admission

• Fever: number of days body temperature ≥38

• Pain score: number of days Numeric Rating Score ≥7

• Hospital infections: infusion-, urinary track-, airway-, and postoperative wound infections

• Presentation at department of emergency, within one month after discharge

• Non-elective readmission within one month after discharge

Process indicators:

• Days between discharge and letter of discharge

• Introduction hospitalist to the patient less than 24 hours after hospital admission

Data about unplanned readmission and presentation at emergency department after discharge are collected using self-administered patient questionnaires, which are send at one month after discharge date. Information about the other indicators will be retrospectively derived from patient medical records.

#### ***Patients health-related quality of life***

Generic health-related quality of life is measured with the Euroqol-5D (EQ-5D), which is a widely used validated questionnaire containing five domains: mobility, self-care, usual activities, pain, and anxiety/depression [23]. Each domain has three possible levels indicating; no problems, moderate problems or sever problems. Besides, respondents are asked to value their overall health status on a visual analog scale, ranging from 0 (defined as the worst imaginable health state) to 100 (defined as the best imaginable health state). The EQ-5D is assessed by patient questionnaires at three time points: at admission, discharge and one month after discharge.

#### ***Patient experiences with medical ward care***

Patient experiences with medical ward care are assessed by a self-administered questionnaire at discharge. This questionnaire focuses on satisfaction with communication, experienced continuity of care and cooperation, and the patients view on the medical competencies of the hospitalist. Patient perceptions on communication skills of the hospitalist are measured with the Communication Assessment Tool (CAT), which consists of 15 questions and can be rated on a 5 point Likert scale, ranging from ‘poor’ to excellent’. Although not validated in the Netherlands, the CAT has already proven to be a reliable and valid instrument in the hospital setting in the US [24]. Three subscales from the ‘Chronically Ill Patients Evaluate general Practice’ (CEP) questionnaire were added to measure the items satisfaction with continuity of care, cooperation of ward care providers, and medical competencies of the hospitalist [25]. Each item will be rated on a six point Likert scale, ranging from ‘poor’ to ‘excellent’. As this questionnaire has only been validated for primary care, psychometric properties will be examined in this study. To ensure that patients know who their hospitalist is, we include photos from the hospitalist(s) in the questionnaire. To assess whether patients understood the questions asked in the self-administered questionnaires, we pre-tested the questionnaire in a sample of ten patients admitted to two hospital wards in different hospitals.

#### ***Health professionals’ work experiences and job characteristics***

An online questionnaire is compiled to measure job satisfaction, distress outcomes and other job characteristics of the care providers working at the included hospital wards; i.e. all MDs and PAs who fulfill the role of hospitalist, and a random sample of ten nurses in each of the participating wards.

*Job satisfaction* is assessed with the McCranie Job Satisfaction Scale, which consists of 13 questions which can be rated on a 6-point Likert scale, ranging from very dissatisfied to very satisfied [26]. The questionnaire addresses satisfaction with the amount of time which is available per patient, satisfaction with the level of work challenge, and satisfaction with the collaboration with nurses, medical specialists and medical residents. Some items were rephrased to make them appropriate for the specific profession of our interest and some questions were added. For all professions we additionally ask about satisfaction with collaboration with PAs. Besides, in the questionnaire for medical specialists a question about satisfaction with time spend on supervision was added, and in the questionnaire for hospitalists we additionally ask for satisfaction with the received supervision. Finally, respondents are asked to value their overall job satisfaction on a visual analog scale, ranging from 1 (extremely unsatisfied) to 10 (extremely satisfied).

*Job stress* is assessed by the 12-item General Health Questionnaire (GHQ-12). The GHQ-12 is a unidimensional, validated scale which comprises questions regarding anxiety, depression, social dysfunction, and loss of confidence. Statements are rated on a 4-point rating scale (symptom present: “not at all” = 0, “same as usual” = 0, “more than usual” = 1, and “much more than usual” = 1) GHQ-12 scores range from 0 to 12 with a higher score indicative of poorer psychological well-being [27].

*Workload* of hospitalists is measured in terms of number of patients seen per day and weekly overtime hours. We ask hospitalists (both PAs and medical residents) and medical specialists for the number of hours per week spend on both direct and indirect patient contacts at the hospital ward, and the number of hours per month spend on non-patient related tasks like participating in quality and patient safety projects and performing scientific research. In the questionnaire for hospitalists we additionally ask for the number of hours spend on patient related non-hospitalist tasks like performing medical procedures or supporting outpatient care. Besides, we ask the hospitalists how much supervision time they obtain, and the medical specialists how many time they spend on supervision.

#### ***Continuity of care***

Effects of substitution of hospital ward care on patient experienced continuity of care are measured by a set of questions in the patient questionnaire at discharge, as described in the section ‘patients experiences with medical ward care’. Additionally, continuity of care is established by evaluating work schedules. All hospitalists are asked to fill in their real work schedule during fixed weeks: week 3, 7, 11 and 15 after the start of the inclusion of patients. Continuity of care will be assessed by counting the number of rotations in the hospitalist position during these fixed four weeks. Data collection runs parallel for each pair of matched intervention and control ward.

### Qualitative research

Semi-structured interviews are conducted to identify determinants which contribute to the safety, clinical effectiveness and cost-effectiveness of hospital ward care by PAs. Also barriers and facilitators for the implementation of PAs in the role of hospitalist are explored. The interviews are held with a sample of PAs, (specialized) MDs, heads of the departments and nurses. Sampling is done purposively. A variety of care providers are include, covering different medical specialties and medical ward care models. Interviews will be taken until data saturation is achieved on the basis of interim-analyses after each set of five to eight interviews, with a minimum of twenty interviews. A topic list, which will be refined iteratively during the process of data collection and analysis, is used to frame the interview. The TICD framework of Flottorp et al is used to standardize the reporting of barriers and facilitators [28]. Barriers are analyzed in the context of the innovation itself, the individual professional and the patient, and the social context, the organizational context and the economic and political context.

### Economic evaluation

To assess the cost implications and efficiency of substitution of hospital ward care from MDs to teams with PAs, an economic evaluation is conducted alongside the outcomes evaluation. This economic evaluation is based on the general principles of a cost-effectiveness analysis, except that the time horizon per included patient is limited to one month after discharge. If equivalence of effects is established the economic decision rule alters in ‘cost minimization’. The primary cost outcome for the economic evaluation is costs associated with the principal admission (LoHS, resource use, consultation of health care suppliers, salaries, productivity loss) and costs that occurred after discharge that is potentially related to hospital ward care (unplanned readmission, presentation at emergency departments, visits of general practitioner, required home care, productivity loss) in a period from admission until one month after discharge (Table 1). The primary effect outcome in the economic evaluation is EQ-5D based QALYs. We will also analyze costs in relation to LoHS, the primary outcome in the outcomes evaluations. Besides these costs and effects, information about patient characteristics such as gender, age, primary diagnoses and co-morbidities are collected in order to account for patient case-mix as far as possible. All patient-related volumes are collected in detail at an individual patient level, primarily from medical patient records and patient and care provider questionnaires. Costs will be calculated by multiplying the volumes of healthcare use with corresponding unit prices, derived from the Dutch Manual for Costing Research [29], which also include organizational overhead costs. All figures will be related to the price level of the same year.

**Table 1:** Volumes included in the economic evaluation

**Volume**	**Unit**
During hospital stay at the included ward^*^	
Length of hospital stay	Number of days
Non-elective transfer to ICU	Number of days
*Resource* use:	
Surgery	Type of surgery
Medication	Frequency, dose and type of medicine
Laboratory tests	Frequency and type of blood test
Radiographic imaging	Frequency and type of radiographic imaging
Scopic tests	Frequency and type of scopic test
Blood components	Number of units
Consultation with health care suppliers^‡^	Number of consultations
*Medical ward staff:*	
Hospitalists	Working hours per week hospitalist
Supervision by medical specialist	Number of hours supervision per week
**During the first month after discharge**^†^	
Non-elective presentation at emergency department after discharge	Number of presentations at emergency department
Non-elective readmission	Number of days
Non-elective visit to GP	Number of visits to GP post
	Number of visits by GP at patient’s home
	Number of visits to GP
	Number of telephone contacts with GP
Required nursing home care	Number of hours per week
Required domestic home care	Number of hours per week
Productivity loss	Hours per week

*Abbreviations*: *ICU* Intensive Care Unit, *GP* General Practitioner.

^*^Assessed by extraction of patient medical records.

^†^Assessed by patient questionnaires one month after discharge.

^‡^e.g. medical specialist, physiotherapist, dietician, diabetes nurse, occupational therapist, medical social work, psychologist.

### Confounders

Because of the non-randomized character of this study and the heterogeneity of patients and hospital wards, there is a risk of confounding. We will correct for a number of predefined confounders in the statistical analyses. The covariables related to *patients* are: gender, age, education, ethnicity, marital status, smoking status, body mass index, primary diagnosis, co morbidities, number of prior hospitalizations, type of admission (elective or emergent), discharge destination and the health-related quality of life at admission. *Healthcare provider factors* are gender, age, highest education, profession, years since graduation, years on the job, extent of employment, regularity of work schedules and workload. *Hospital ward characteristics* are medical specialism, hospital type, teaching status, number of admissions, bed occupancy, and number of MDs, PAs and nurses are assessed. Covariables are extracted from patient medical records and patient and care provider questionnaires.

### Sample size calculation

To detect a relative difference in LoHS of 20% between the mixed ‘PA/MD model’ and solely ‘MD model’, assuming an average LoHS of 7 days [30], alpha 5%, power 80% and an ICC of 0.06 for patients in same ward, 40 wards including 100 patients each are required. Taking into account an expected drop-out rate of 10% at the level of wards, and a 10% drop out rate of patients (withdrawal of informed consent), 44 wards (22 in each arm) with each 115 patients are included. The number of in depth interviews depends on the moment data saturation is attained.

### Data analyses

To compare hospital wards utilizing a mixed ‘PA/MD model’ with wards utilizing a solely ‘MD model’, we use logistic regression analyses for dichotomous outcomes and linear regression analysis for continuous outcomes, both with random coefficients to account for statistical clustering of data in hospital wards. The analysis is on an intention to treat basis and matching will be taken into account. Missing values are substituted by multiple imputation techniques. Multivariable models are constructed to correct for potential confounders. Covariables are included in the final model only if they modify the regression coefficient of ward care model (i.e. the central determinant) by more than 10% (regardless of statistical significance of effects). Explorative subgroup analyses per medical specialism will be conducted for each set of at least six wards with similar specialism are included. All estimates are calculated with 95% confidence intervals.

### Economic analyses

Discounting of costs and effects is applied as recommended for health economic evaluations in The Netherlands [29]. A comparison is made between the intervention and control group on incremental costs and incremental effects. The incremental cost-effectiveness ratio (ICER) will be calculated as follows: ICER = (Δ costs/Δ effects) where Δ costs represents the difference in annual mean costs between intervention and control group, and Δ effects represents the difference in QALYs between the two groups.

The uncertainty associated with estimates is explored with a bootstrap resampling procedure to produce cost-effectiveness planes as well as targeted one-way sensitivity analyses of potential drivers of key cost (such as type of ward). The bootstrapped ICERs will be presented in a cost-effective acceptability curve displaying the probability that the intervention is cost-effective for a wide range of willingness-to-pay thresholds. P-value is set at 0.05 to indicate statistical significance. To test for several assumptions (i.e. cost-prices and salary), one-way sensitivity analyses will be conducted on the range of extremes.

### Qualitative data analyses

The semi-structured interviews are audio-taped and transcribed verbatim with participants consent. A deductive process of thematic analysis is used to classify responses within themes. The theoretical domains previously described are used as the coding framework. Analyses are conducted in Atlas.ti software. Two researchers will code and analyze the transcript independently to reduce subjectivity. Consensus is reached by discussion. Member checking confirm the credibility of the data: each participant will be given a full transcript of the interview with a summary of themes to determine whether the themes were appropriately identified and matched their responses.

### Ethical considerations

The research ethics committee of the Radboud university medical center has declared that this study doesn’t fall within the remit of the Medical Research Involving Human Subjects Act (WMO) (registration number 2012/306). This means that this research can be carried out without an approval by an accredited research ethics committee. All data will be handled strictly confidential. Written informed consent is obtained from all patients.

## Discussion

To our knowledge, this is the first multicenter study which investigates the efficacy and effectiveness of reallocation of hospital ward care from MDs to PAs. Most international studies on reallocation of care to PAs are restricted to primary or critical care, limited to one outcome measure, or are of insufficient methodological quality [2].

The major strengths of this study are the multicenter design and the broad view; we perform measurements both at patient, care provider and hospital ward level. A wide variation of instruments and methods is used to obtain data; we use both quantitative measurements (medical patient records, patient and care provider questionnaires, work schedules) and qualitative measurements (semi-structured interviews). As a consequence, we provide not only useful information about the objective effects of reallocation of hospital ward care on a range of outcomes, but we are also able to determine barriers and facilitators for the implementation.

One of the limitations is the non-randomized design of this study. In the Netherlands, PAs followed a so-called ‘dual program’, which means that students are employed within a particular medical specialty while enrolled in the master’s PA program (Table 1). After graduation, PAs are intended to be employed at the same department. The suggestion of randomly relocating the graduated PA to other hospital wards would lead to resistance among the medical specialists who put considerable effort and time to training and supervision.

The non-randomized character of this study implies an increased risk for confounding, which we will take into account in the multivariable analyses. Another challenge is to deal with the heterogeneity of patients across hospital wards. Each hospital differs slightly in determinants like the organization of ward care (care by medical resident or specialist, arrangement of supervision), policies about quality of care, patient case-mix and medical subspecialties, which might reduce explained variation and subsequently reduce the power of this study. When appropriate, we will conduct explorative secondary quantitative and qualitative analyses to explain heterogeneity.

This multicenter study adds to the current body of knowledge by creating more knowledge of the effects of task reallocation in hospitals on the efficiency, quality and continuity of care. Findings from this study will help to further define the role of nonphysician clinicians and provides possible key components for the implementation of PAs in hospital ward care.

## Competing interests

MW and MGH Laurant have no conflicts of interest. Both MJC Timmermans and JAH van Vught work as a teacher at one of the PA Master programs.

## Authors’ contributions

ML and MT are responsible for the design of the study with comments of AvV and MW. MT wrote first draft of the manuscript and all other authors revised this critically. MT is responsible for the data collection and data management with direct supervision and feedback from ML. All authors read and approved the final manuscript.

## Pre-publication history

The pre-publication history for this paper can be accessed here:

http://www.biomedcentral.com/1472-6963/14/43/prepub
