# Erratum to: The effectiveness of substitution of hospital ward care from medical doctors to physician assistants: a study protocol

**DOI:** 10.1186/s12913-016-1330-9

**Published:** 2016-04-04

**Authors:** Marijke J. C. Timmermans, Anneke J. A. H. van Vught, Michel Wensing, Miranda G. H. Laurant

**Affiliations:** Radboud University Medical Center, Radboud Institute for Health Sciences, Scientific Center for Quality of Healthcare (IQ healthcare, Nijmegen, The Netherlands; HAN University of Applied Sciences, Faculty of Health and Social Studies, Nijmegen, The Netherlands

Unfortunately, the original version of this article [1] contained an error in the text. The correction of this error and also an adjusted sample size calculation is detailed below.

## Corrections

### Year of authorization of PAs

After publication of our study protocol, we noticed an error at the fifth bullet on page 2 [1]. We described that since January 2013 PAs are authorized to indicate and perform predefined medical procedures and subscribe medication without supervision. January 2013 should however be January 2012 [2].

### Adjusted sample size calculation

In the original study protocol we described a sample size calculation in which an average length of hospital stay (LoHS) of 7 days and a standard deviation of 6 days was used. These numbers were based on a study of Borghans et al, in which the LoHS was presented of all patients who were admitted at 69 hospitals in the Netherlands during one year [3]. This concerned all possible medical specialisms. However, we included the following specialisms in our study population: general surgery, pulmonology, gastroenterology, cardiology, orthopedics and otolaryngology (ENT). The medical specialisms with relatively high LoHS (f.e. cardiothoracic surgery, geriatrics, dermatology) and relatively low LoHS (f.e. ophthalmology, plastic surgery, gynecology) were not represented [4]. This composition prompted us to recalculate the required sample size. Instead of a LoHS of 7 days and a SD of 6, a LoHS of 6 days and a SD of 4.8 days was used, which better fitted with our study population. All other parameters remained the same. Taking into account an expected drop out of maximum 2 matched pairs, 34 wards (17 in each arm) with each 100 patients are required. In case of no drop out, 50 patients per ward are sufficient to detect a significant difference in LoHS, with an expected 20 % reduction in LoHS, alpha 5 %, power 80 % and ICC 0.06.

As a consequence of the matched controlled study design, the SD in our study population might be smaller than the above mentioned SD of 4.8, but we are unable to provide reliable estimates [5].
